# Structural Analysis, Multi-Conformation Virtual Screening and Molecular Simulation to Identify Potential Inhibitors Targeting pS273R Proteases of African Swine Fever Virus

**DOI:** 10.3390/molecules28020570

**Published:** 2023-01-06

**Authors:** Gen Lu, Kang Ou, Yihan Zhang, Huan Zhang, Shouhua Feng, Zuofeng Yang, Guo Sun, Jinling Liu, Shu Wei, Shude Pan, Zeliang Chen

**Affiliations:** 1 Key Laboratory of Livestock Infectious Diseases, Ministry of Education, Shenyang Agricultural University, No. 120, Dongling Road, Shenhe District, Shenyang 110866, China; 2 The Preventive and Control Center of Animal Disease of Liaoning Province, Liaoning Agricultural Development Service Center, No. 95, Renhe Road, Shenbei District, Shenyang 110164, China; 3 Qianyuanhao Biological Co., Ltd., Building 20, District 11, No. 188 South Fourth Ring West Road, Fengtai District, Beijing 100070, China

**Keywords:** African swine fever, antiviral drugs, molecular dynamics simulation, multi-conformation virtual screening

## Abstract

The African Swine Fever virus (ASFV) causes an infectious viral disease in pigs of all ages. The development of antiviral drugs primarily aimed at inhibition of proteases required for the proteolysis of viral polyproteins. In this study, the conformation of the pS273R protease in physiological states were investigated, virtually screened the multi-protein conformation of pS273R target proteins, combined various molecular docking scoring functions, and identified five potential drugs from the Food and Drug Administration drug library that may inhibit pS273R. Subsequent validation of the dynamic interactions of pS273R with the five putative inhibitors was achieved using molecular dynamics simulations and binding free energy calculations using the molecular mechanics/Poison-Boltzmann (Generalized Born) (MM/PB(GB)SA) surface area. These findings demonstrate that the arm domain and Thr159-Lys167 loop region of pS273R are significantly more flexible compared to the core structural domain, and the Thr159-Lys167 loop region can serve as a “gatekeeper” in the substrate channel. Leucovorin, Carboprost, Protirelin, Flavin Mononucleotide, and Lovastatin Acid all have Gibbs binding free energies with pS273R that were less than −20 Kcal/mol according to the MM/PBSA analyses. In contrast to pS273R in the free energy landscape, the inhibitor and drug complexes of pS273R showed distinct structural group distributions. These five drugs may be used as potential inhibitors of pS273R and may serve as future drug candidates for treating ASFV.

## 1. Introduction

African swine fever (ASF) is an acute, highly infectious disease in pigs caused by the African swine fever virus (ASFV). Its clinical symptoms vary in the acute stage, with a case fatality rate of 100%. The disease is extremely harmful to the pig industry and must be reported to the World Organization for Animal Health [1]. The first case of ASF in China was reported on 3 August 2018. Thereafter, the disease spread rapidly to various provinces, municipalities, and autonomous regions across the country. Owing to the lack of preventive vaccines and treatment drugs, ASF poses a continuous threat to the stable development of the pig industry in China [2,3].

The pS273R protease is a key target protein for designing and developing effective inhibitors of ASFV. ASFV pS273R protease, a novel SUMO-1 specific protease, is synthesized and localized in the cytoplasmic viral plant during the late stages of viral infection [4]. P220 and p62, encoded by the ASFV genes CP2475L and CP530R, are polyprotein precursors that make up the ASFV core-shell. P220 and p62 are cleaved by intrinsic pS273R protease hydrolysis, producing p5, p14, p34, p37, and p150 from p220 and p8, p15, and p35 from p62, respectively, which are essential for the maturation and infectiousness of ASFV [4,5]. The Crystal structure of pS273R was elucidated in 2020. It consists of two domains, the N-terminal arm domain and the core domain. The core domain contains residues N84 to A273, and has high structural similarity to chlamydial deubiquitinase, sentrin-specific protease, and adenovirus protease [6]. The protease core domain contains conserved catalytic cysteine and histidine residues, and the active site pocket is a triad of residues cut from a directed catalytic substrate (His168-Asp187-Cys232). The arm domain comprises residues M1 to N83, that are unique to ASFV and play an important role in maintaining the enzymatic activity of ASFV pS273R [6]. 

The structural differences between pS273R and other proteases make pS273R an attractive target for the development of drugs against ASFV. Indeed, previous work has shown that reduced expression of pS273R inhibited polyprotein processing, and significantly reduced infectious virus production [6,7,8]. In addition, researchers have found that gasdermin D (GSDMD) can also be cut by ASFV pS273R. The pS273R interacts with GSDMD at Gly107 and cuts it, suggesting that pS273R can break down GSDMD to regulate apoptosis [9]. Additionally, pS273R can also inhibit the production of genes stimulated by type I interferon signaling, suggesting that pS273R may interact with and cut the core components of the interferon signaling pathway and disrupt the interferon response. In summary, pS273R, a cysteine protease, performs a critical role in the ASFV lifecycle. The pS273R protease is an extremely attractive target for the design and development of effective inhibitors for ASFV therapy, similar to drug development programs for HIV and SARS-CoV2 proteases [10,11].

Despite ongoing efforts, there are currently no effective drugs for the treatment of ASF, several molecules have been identified as having inhibitory activity against the pS273R protein of the ASFV. These include a group of peptide inhibitors based on the human SUMO-1 substrate complex (RCSB_PDB: 1TGZ) that contain an electrophilic warhead (-CHO), and the defensin-like peptide toxin OPTX-1 and the small molecule inhibitor E64 [7,12]. The peptide inhibitor taking the CHO group contains a carbonyl carbon, which is an electrophilic group capable of attracting electrons from nucleophilic groups. Similarly, the epoxide carbon of the E64 molecule is also an electrophilic group. Once these molecules interact with the mercapto group of CYS, electrophilic attack occurs, resulting in the formation of covalent bonds between the inhibitor and pS273R, this binding prevents the hydrolytic activity of pS273R. Inhibitors that form covalent bonds with their targets have traditionally been considered highly adventurous due to their potential off-target effects and toxicity concerns [13,14]. It has been observed that OPTX-1 inhibits pS273R through non-covalent interactions, indicating the possibility that non-covalent inhibitors may also be effective in inhibiting pS273R [12]. However, there is a need for further exploration of potential drugs for pS273R in order to effectively combat ASFV. Structure-based virtual screening (SBVS) is a commonly used technique in modern drug discovery [15,16,17,18], and molecular docking has been applied in the identification and design of antiviral drugs for a variety of animal viral diseases, including ASFV, porcine reproductive, and respiratory syndrome virus (PRRSV) and Porcine epidemic diarrhea virus (PEDV) [19,20,21].

Molecular dynamics (MD) simulations has been demonstrated to be a valuable technique for sampling protein conformations [22], and the combination of molecular simulation and molecular docking has been a popular approach in this regard [23,24]. The identification of inhibitors for pS273R requires the examination of different conformations of the flexible ring (THR159-LYS167), and it has been shown that considering this conformational ensemble leads to better results than using single crystal structures [22,25,26]. As such, this study aimed to explore the conformational space of pS273R through MD simulation. MD simulations of pS273R were conducted for 200 ns, and representative structures were extracted through clustering of the simulation trajectories for use in molecular docking. The use of multiple scoring functions further enhanced the performance of the virtual screening process [27], and the candidate molecules identified through this process were subsequently validated using MM/PB(GB)SA, providing new chemical starting points for the development of pS273R inhibitors.

## 2. Results

### 2.1. Conformation Sampling and Structure Analysis

**Figure 1:**
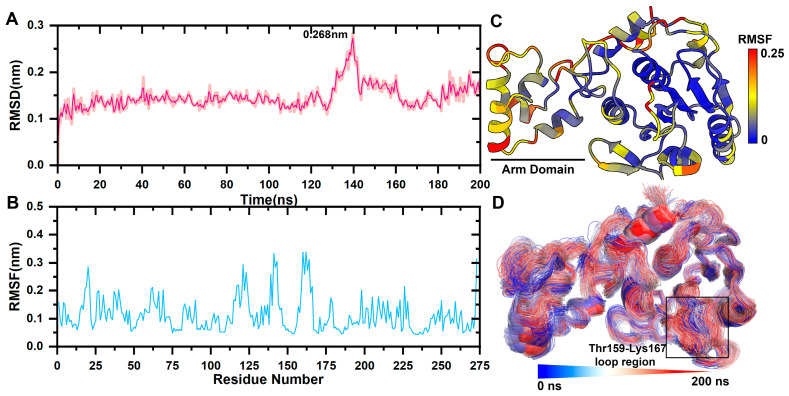
pS273R Molecular dynamics simulation. (**A**) Molecular kinetics of RMSD values for long-scaffolded carbon atoms of pS273R over 200 ns. (**B**) Molecular kinetics of RMSF values for amino acids of pS273R over 200 ns. (**C**) RMSF at 200 ns projected onto the pS273R structure. (**D**) Molecular dynamics simulation trajectory of pS273R from 0 to 200 ns. Note: Black box means the loop region of Thr159-Lys167 of pS273R.

The mean square root deviation (RMSD) was used to determine the average deviation of the protein conformation from the original conformation at a given time and to assess whether the complex system had reached a stable state. The backbone C atom of the free pS273R protein experienced a brief initial increase in RMSD, followed by a narrow oscillation state, and peaked at 0.268 nm after 120 ns. It was the result of Thr159-Lys167 movement in the arm domain and loop region. Subsequently, the RMSD quickly fell back within 0.2 nm (Figure 1A).

The mean square root fluctuation (RMSF) measures the degree to which the residue fluctuates across a trajectory relative to its average position. RMSF values was projected onto the tertiary structure of the protein. The catalytic triplets His168, Cys232, and Asn187 in the core domain are responsible for the catalytic activity of cysteine enzymes and are highly conserved. His168, Cys232, and Asn187 have a lower RMSF, and the arm domain had a higher RMSF value than the core domain, indicating that it was more relaxed in its physiological state (Figure 1B,C).

Catalytic triplets form a pS273R active pocket with the Thr159-Lys167 loop region, and the area is very flexible. The initial conformation was fitted to the simulated tracks (Figure 1D). The arm domain with the Thr159-Lys167 loop region experienced the most pronounced movement.

**Figure 2:**
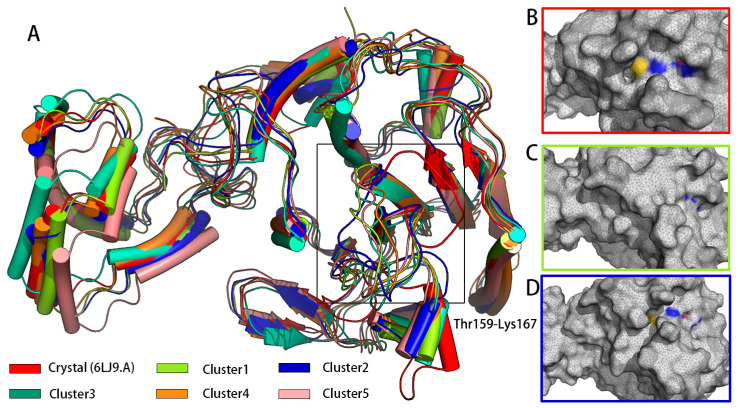
The main conformation extracted from pS273R in 200 ns simulation duration. (**A**) Crystal structure of five major conformation clusters in pS273R. Note: Black box means the loop region of Thr159-Lys167 of pS273R. (**B**) Surface structure of pS273R Crystals. (**C**) Surface structure of Cluster 1. (**D**) Surface structure of Cluster 2. Note: Oxygen atoms of His168, Cys232, and Asn187 residues are red, nitrogen atoms are blue, and sulfur atoms are yellow.

**Figure 3:**
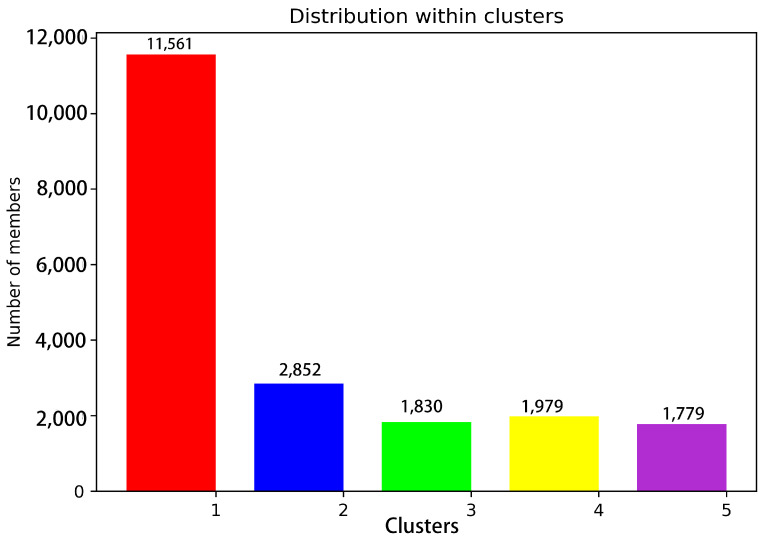
Statistics of 20,000 snapshot species per conformation cluster at 200 ns. Note: Red represents Cluster 1; blue represents Cluster 2; green represents Cluster 3; yellow represents Cluster 4; purple represents Cluster 5.

Conformation clustering can be used to easily extract meaningful information from the trajectory of a MD simulation from a copolymerization class to five conformation clusters. Consistent with what was observed in the RMSF, fitting the clusters on pS273R Crystal (6LJ9A) revealed pronounced movement of the arm domain and Thr159-Lys167 loop region, with conformation and Crystal (6LJ9A) calculated to have RMSD values of 1.025, 1.439, 1.300, 1.591, and 1.591 nm, respectively (Figure 2). Cluster 1 and Cluster 2 accounted for more than 72% of configurations, and thus may represent the primary conformation of pS273R in its physiological state. When the surface structure of pS273R was investigated, Crystal (6LJ9A) had the most significantly active pocket, exposing the catalytic triad to solvents. The catalytic triad of Cluster 1 was encased internally, while the catalytic triad of Cluster 2 was exposed, but the cavity was not as open as the Crystal structure (Figure 3), Cluster 2 appears to be situated between Cluster 1 and Crystal (6LJ9A), indicating that dynamic changes in the THR159-LYS167 ring region regulate the opening and closing of the active pocket. Preliminary determination of the Thr159-Lys167 loop region controls the opening and closing of the pS273R catalytic pocket.

**Figure 4:**
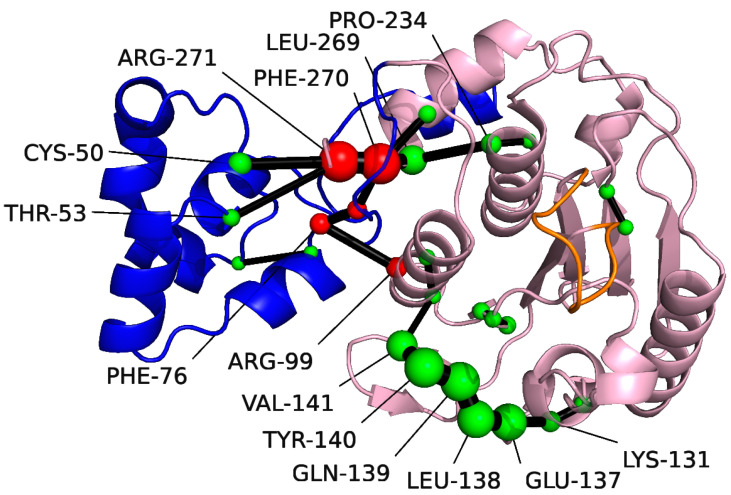
Amino acids identified with the Shortest Path Map (SPM) for pS273R using Sphere. The Arm structural domain colored in blue, the core structural domain in pink, and THR159-LYS167 in orange. The size of the sphere indicates the importance of the location, and the black edge indicates the communication pathway (how the different residues are connected). Residues at the communication interface between the Arm region and the core structural domain colored in red.

The shortest path analysis was performed on pS273R, which can further understand the pS273R protein conformational movement, as shown in Figure 4, two major allosteric pathways of the ARM remote regulatory core region were found, Arg271, Phe270, Leu296, and Pro234 are important residues for the motor communication between ARM region and core region. Notably, Pro234 is located near the catalytic residue Cys232. Another allosteric pathway shows key residues in the motor communication of Phe76 and Arg99. In addition, Lys131-Val14 located in the core region showed strong motor correlation.

### 2.2. Virtual Screening and Calculation of MDockScore

Crystal (6LJ9A), Cluster 1, and Cluster 2 were selected as the receptors for virtual screening (Table 1). Nineteen molecules had watvina docking scores below −6.5 kcal/mol for Crystal (6LJ9A), Cluster 1, and Cluster 2 (Table 1). Among Crystal (6LJ9A) configurations, configuration 2921-57-5 had the highest docking score at −9.3 kcal/mol. For Cluster 1, configurations 146-17-8 and 75225-51-3 had the highest score at −7.3 kcal/mol. For Cluster 2, configuration 27025-41-8 had the highest score at −8.4 Kcal/mol.

The score function can significantly reduce the false positivity rate and improve the results of virtual screening. Calculation details and weights are shown in Supplementary Table S1. Leucovorin was found to have the highest MDock score at 2.911. Carboprost, Protirelin, Flavin Mononucleotide, and Lovastatin Acid had MDock scores of 2.317, 2.243, 2.174, and 2.154, respectively (Table 2).

**Figure 5:**
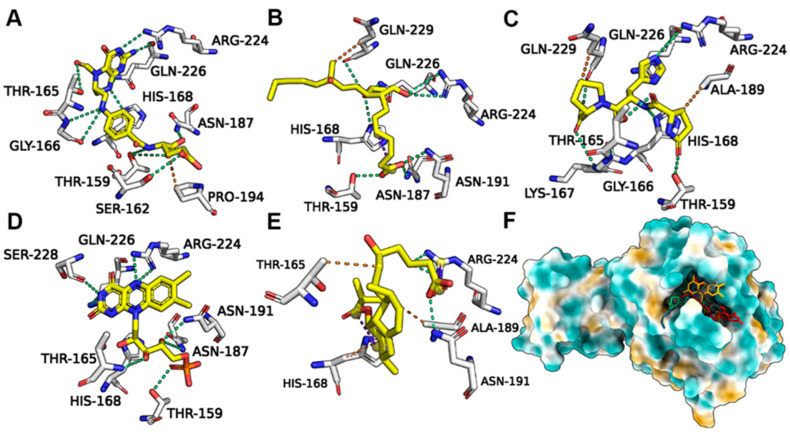
Binding conformations of FDA-approved drugs with ASFV pS273R. (**A**) Leucovorin with Crystal (6LJ9A). (**B**) Carboprost with Crystal (6LJ9A). (**C**) Protirelin with Crystal (6LJ9A). (**D**) Flavin Mononucleotide with Crystal (6LJ9A). (**E**) Lovastatin Acid with Crystal (6LJ9A). (**F**) Positions of the five candidate molecules in the Crystal (6LJ9A) active pocket.

Leucovorin underwent hydrogen bond (H-bond) interactions with pS273R TH159, SER162, GLY166, HIS168, ASN 187, ARG224, and GLN226, and hydrophobic interactions with PRO194 (Figure 5A). Carboprost interacted with TH159, HIS168, ASN187, ASN191, ARG224, GLN226, and GLN229 via H-bonds and exhibited hydrophobic interactions with GLN-229 (Figure 5B). Protirelin exhibited H-bond interactions with TH159, GLY166, LYS167, ARG224, GLN226, GLN 229, THR159, HIS168, ASN 187, and ARG224, and hydrophobic interactions with GLN-229 and ALA189 (Figure 5C). Flavin Mononucleotide generated H-bond interactions with TH159, HIS168, ASN187, ARG224, GLN226, SER228, and GLN-229, and hydrophobic interactions with AlA189 (Figure 5D). Lovastatin Acid exhibited H-bond interactions with ASN191, ARG224, HIS168, AlA189, and THR165 (Figure 5E). Leucovorin, Carboprost, Protirelin, Flavin Mononucleotide, and Lovastatin Acid occupied the active pocket of pS273R (Figure 5F).

### 2.3. The Analysis of Molecular Dynamic Simulation Based on the Top Five pS273R–Drug Ligand Complexes

**Figure 6:**
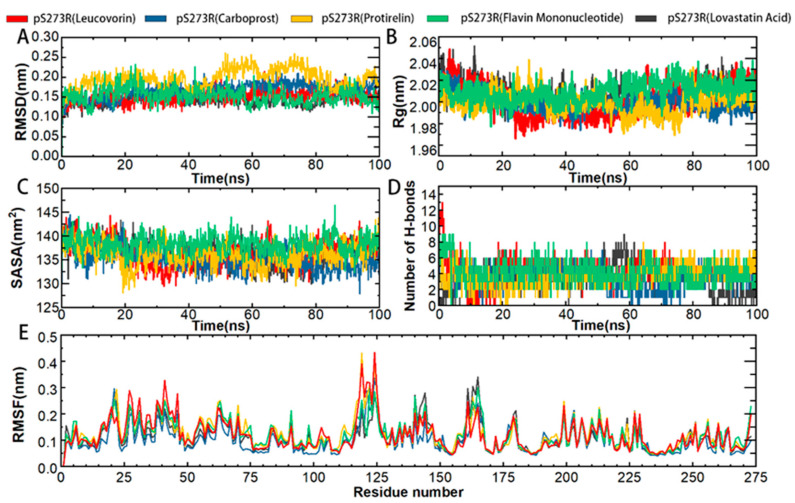
Molecular kinetics simulation of pS273R and its putative drug molecular complex over 100 ns. (**A**) Skeleton Carbon RMSD. (**B**) Rg of pS273R α carbon atoms. (**C**) pS273R α Carbon SASA. (**D**) Number of H-bonds between the drug and pS273R. (**E**) RMSF of pS273R residues.

Further simulations of the complexes of each of the five drugs with pS273R were performed to characterize the binding stability and conformational changes of the pS273R protein products induced by the drug molecules. Protein skeletal RMSD values were calculated as a function of simulated time to elucidate the dynamic stability of the complexes. For potential inhibitors, 100 ns were simulated and analyzed (Figure 6). The pS273R complexes with Leucovorin were the fastest to enter equilibrium, followed by those with Carboprost, Flavin Mononucleotide, Lovastatin Acid, and Protirelin. The pS273R-Leucovorin, pS273R-Carboprost, pS273R-Flavin Mononucleotide, and pS273R-Lovastatin Acid complexes changed during the initial 30 ns of the simulation and stabilized after 50 ns. The pS273R–Proterelin complexes exhibited broad-band oscillations in skeletal RMSD during the first 50–80 ns, reaching a maximum of 0.26 nm, and the RMSD subsequently retreated to approximately 0.20 nm after 80 ns. The simulation results demonstrate that the RMSD of the backbone carbon atoms of the complex reached a stable state after convergence (Figure 6A).

To predict the compactness of the protein-ligand complexes, the Rg (Gyration radius) and solvent accessible surface area (SASA) of the analog system were calculated. Rg is an effective parameter for evaluating the architectural integrity and compactness of a system. Rg is defined as the mass-weighted square root distance between atoms assembled in their common centroid. SASA determines the accessibility of proteins to solvents and is indicative of protein unfolding in MD simulations. The time evolution diagram of Rg shows that all systems are compact. Carboprost made proteins more compact and had lower Rg and SASA values than the other drugs. Carboprost was more stable in combination with pS273R at low Rg and SASA values compared with other drugs. The combination of Flavin Mononucleotide and Leucovorin with pS273R increased the Rg and SASA values, suggesting that these drugs have a potential role in the unfolding of pS273R structures (Figure 6B,C). Overall, the drug combinations resulted in compact changes in pS273R and increased flexibility.

H-bonds non-covalently bind two molecules, and GROMACS provides a more rigorous determination of hydrogen bonds The binding properties of the pS273R protein to a hypothetical inhibitor were analyzed by mapping the number of intermolecular H-bonds over time. The most significant number of H-bonds (up to nine) was recorded in the pS273R-Lovastatin Acid complex after 20 ns of simulation, but they were unstable. Leucovorin, Protirelin, and Flavin Mononucleotide formed more stable H-bonds with pS273R. After 20 ns, Leucovorin, Carboprost, Protirelin, Flavin Mononucleotide, and Lovastatin Acid had average H-bond bars of 4.6, 3.4, 3.8, 3.9 and 3.5, respectively, with the pS273R protein (Figure 6D).

Compared to free pS273R, the binding of Lovastatin Acid did not significantly affect the RMSF of pS273R (Figure 6E). The combination of Leucovorin and pS273R reduced the RMSF in the Gln139-Arg148 and Thr159-Lys167 regions, while increasing RMSF in the Met115-Val130 and arm regions. The combination of Carboprost and pS273R reduced the RMSF in the Gln139-Arg148 and Thr159-Lys167 regions. The binding of Protirelin to pS273R reduced the RMSF in the Gln139-Arg148 and Thr159-Lys167 regions and increased the RMSF in the Met115-Val130 and arm regions. The combination of Flavin Mononucleotide and pS273R resulted in a slight decrease in the RMSF in the Gln139-Arg148 and Thr159-Lys167 regions. From these RMSF results, we conclude that the overall fluctuation of the Thr159-Lys167 region decreased after drug combination, with the most significant decrease in RMSF observed in the pS273R-Carboprost complex (Supplementary Figure S1).

**Figure 7:**
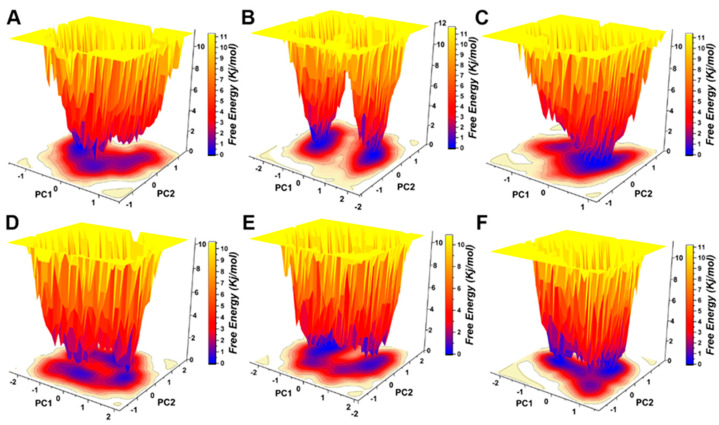
Free energy landscape analysis of the pS273R monomer and hypothetical drug molecular complexes. (**A**) Free pS273R monomer. (**B**) pS273R-Leucovorin complex. (**C**) pS273R-Carboprost complex. (**D**) pS273R-Proterelin complex. (**E**) pS273R-Flavin Mononucleotide complex. (**F**) pS273R-Lovastattin Acid complex.

Protein motility was projected on PC1 and PC2 in the five hypothetical complexes and ionized pS273R simulations (Figure 7A). Dissociated pS273R showed three main conformational spaces. pS273R easily crossed from a low potential to an energy basin with relatively small conformational changes through a low transition potential barrier separation of <2.0 Kj/mol (Figure 7B). Leucovorin exhibited two central conformational spaces separated by potential barriers >8.0 Kj/mol (Figure 7C). Carboprost produced a central low potential energy basin and a low potential energy level that was not easily distinguishable (Figure 7D). Protirelin exhibited three central conformational spaces separated by a low transition potential of <2.0 Kj/mol (Figure 7E). Flavin Mononucleotide showed three central conformational spaces similar to the wandering pS273R conformational spaces (Figure 7F). Finally, Lovastatin Acid exhibited three main conformational spaces. Leucovorin and Carboprost significantly altered the free energy landscape (FEL) map of pS273R, suggesting that these drugs induced different conformation sets of pS273R during the 100 ns simulation (Supplementary Figure S2).

### 2.4. Binding Affinity Calculations Using MM/PB(GB)SA

Using the molecular mechanics/Generalized Born (MM/GBSA) surface area method, the binding free energies of Leucovorin–, Carboprost–, Protirelin–, Flavin Mononucleotide–, and Lovastatin Acid–pS273R complexes were −33.44 Kcal/mol, −31.71 Kcal/mol, −25.55 Kcal/mol, −34.14 Kcal/mol, and −26.58 Kcal/mol, respectively (Table 3). The binding free energy to the pS273R complex (MM/PBSA) was −34.63 Kcal/mol, −28.58 Kcal/mol, −23.84 Kcal/mol, −32.20 Kcal/mol, and −27.01 Kcal/mol, respectively (Table 4). The MM/PB(GB)SA results revealed that Leucovorin and Flavin Mononucleotide had the lowest Gibbs binding free energy with pS273R. The high negative values of ΔG binding indicate good interactions between selected ligands and specific receptors, with van der Waals interactions and the static energy of protein–ligand complexes performing a pivotal role.

### 2.5. Similarity between Candidate Molecules and Known Inhibitors

**Figure 8:**
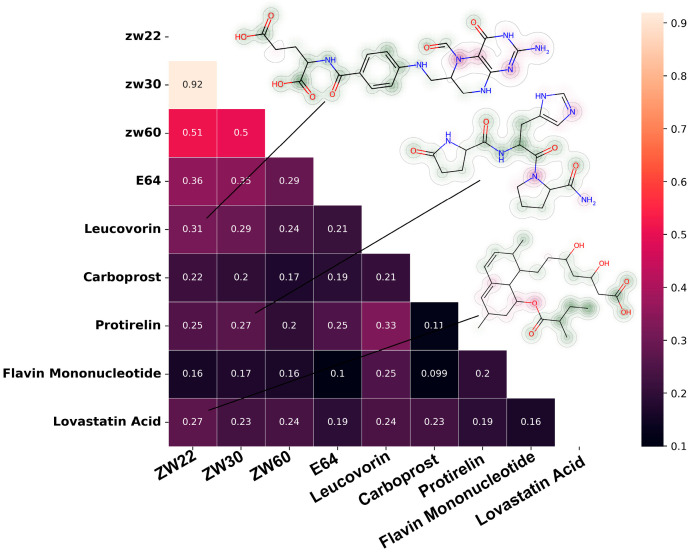
Similarity matrix between candidate molecules and known pS273R inhibitors, the molecule’s fingerprint showed in the upper right corner.

The molecular similarity matrix based on Tanimoto coefficient is shown in Figure 8. The similarity between Leucovorin, Protirelin, and inhibitory inhibitors (ZW22, ZW30) reaches more than 25%, and the similarity between Leucovorin and ZW22 reached 31%. Protirelin showed the highest similarity with E64 (compared with other candidate molecules). Flavin Mononucleotide showed the lowest similarity with inhibitor.

## 3. Discussion

ASFV infection is a major global problem in the pig industry. There are currently no safe or effective drugs or vaccines to treat or prevent ASFV infections [28]. The ASFV pS273R protein is a specific SUMO-1 cysteine protease, distinct from the homologous family of cysteine proteases, whose hydrolysis performs a key role in the maturation and infection of ASFV particles. Therefore, ASFV pS273R is one of the primary drug targets for inhibiting ASFV replication [6]. In addition to its core structure, pS273R contains a unique arm domain that distinguishes it from other members of the SUMO protease family. The pS273R arm domain is unique and contains 83 amino acid residues, it may perform a specific role in maintaining ASFV pS273R activity. The Crystal structure of pS273R and molecular dynamics of the conformational space of pS273R revealed the arm domain is flexible in physiological states. Furthermore, conformation clustering revealed that the catalytic triplet of Cluster 1 was encased internally. Conversely, the catalytic triplet of Cluster 2 was exposed, but the cavity was not as open as the Crystal structure. This indicates that the Thr159-Lys167 loop region controls the opening and closing of pS273R catalytic pockets, acting as a “gatekeeper”. We hypothesized that the movement of the arm domain is likely to be transmitted to the core domain. As analyzed by SPM, there is a regulatory relationship between the ARM domain and the core domain, and residues, such as Arg271 and Phe270, may be the key nodes of the movement network of pS273R residues. In fact, when the C and N terminals of pS273R is truncated, the hydrolytic activity of pS273R is lost [6], and distal regulation of this active site is not uncommon in enzyme catalytic mechanisms [29].

Peptide inhibitors and small molecule inhibitors, such as E64, can inhibit the hydrolase activity of ASFV pS273R [6,7], however, their safety and off-target effects have not been fully demonstrated, therefore, they have not yet been used in clinical trials or entered mass production. In addition, the off-target effects of these inhibitors may impair the function of other SUMO-1 proteases in the cell to some extent. 

Leucovorin (also known as 5-methyltetrahydrofolic acid or calcium folate) is a 5-methyl-derivative of tetrahydrofolic acid and is an essential cofactor in the body. Leucovorin is usually administered in combination with methotrexate (MTX) as a rescue agent to reduce MTX-induced toxicity [30]. Carboprost is a metabolically stable synthetic prostaglandin F2α that binds to the prostaglandin E2 receptor causing uterine muscle contractions, leading to induced labor, or placental discharge [31]. Carboprost is used to treat postpartum bleeding caused by incomplete uterine contractions and terminations in the third trimester. Protirelin is a highly conserved neuropeptide and a synthetic analog of the endogenous peptide thyroid-stimulating hormone release hormone (TRH) [32]. Protirelin stimulates the release of thyroid-stimulating hormone (TSH) and prolactin from the pituitary frontal lobes and regulates TSH levels and neuromodulation. Flavin Mononucleotide is a form of riboflavin (vitamin B2) in which the perhydroxyl group is converted to dihydroxy phosphate [33]. Flavin Mononucleotide is used to restore riboflavin in anemia, migraines, alcoholism, and hypercysteinemia. Lovastatin acid is a powerful monoacetyl-glutarate coenzyme A reductase inhibitor that lowers blood cholesterol and LDL (Low Density Lipoprotein)cholesterol, and thus contributes to the prevention of atherosclerosis and coronary heart disease [34].

MD simulations reveal the kinetic behavior of complex protein motion and catalysis, and how their ligands or inhibitors bind and interact [35]; therefore, MD simulations can identify small-molecule drug candidates for diseases [36]. Such computational tools have emerged as crucial resources in contemporary drug design and bio molecular functional research owing to their lower cost, and time and labor involved. Virtual screening and MD simulations are time- and money-saving methods of repurposing drugs that have been extensively in various disease treatments. Herein, the FDA drug library was used as a starting point for drug discovery. For virtual screening of various protein conformations, pS273R was chose to analysis the main conformation. The accuracy of screening is increased by combining different AI-based receptor–ligand scoring functions. This polymorphic virtual screening method can possibly avoid the potential negative effects of selected inhibitors on other SUMO-like proteins and significantly lower the false positive rate.

The multi-conformation virtual screen result discovered 19 molecules with docking scores < −6.5 Kcal/mol for the pS273R protein polymorphism. These molecules displayed lower score intervals (the lower the better) with pS273R Crystal (6LJ9A), suggesting that the candidate molecules interacted most with Crystal (6LJ9A), with the most open pockets. However, per the MD result, this may not be the predominant pS273R conformation in the physiological state; therefore, even with a larger loop region surrounding the active pocket, this approach is likely to facilitate the drug discovery process for such target proteins. Although the Watvina docking scores of candidate molecules with Cluster 1 and Cluster 2 were lower than those with Crystal (6LJ9A), the Watvina docking scores of all three conformations were <−6.5 Kcal/mol, indicating their relatedness to the Crystal of pS273R, and a certain affinity for Cluster 1 and Cluster 2.

By combining the MDock scores from various scoring functions, Leucovorin, Carboprost, Protirelin, Flavin Mononucleotide, and Lovastatin Acid demonstrated significant potential to inhibit pS273R compared to other molecules. These top five compounds with good MDock scores initially had the highest binding stability to the multiple conformations of pS273R, stable interaction of candidate drug molecules with pS273R is required for pS273R inhibition. By simulating hypothetical binding of inhibitors to pS273R using the RMSD, the induced conformational changes and established the degree of convergence of the analog system are characterized.

The overall structure of the complex remained stable when Leucovorin, Carboprost, Flavin Mononucleotide, or Lovastatin Acid were bound to pS273R. Protirelin binding to pS273R likely results in localized changes in the protein conformation, with wider oscillations observed in Rg and SASA. Flavin Mononucleotide and Leucovorin have potential unfolding effects on proteins. All five molecules above decreased the RMSF value of the Thr159-Lys167 loop region in comparison to the free pS273R. It is hypothesized that the Thr159-Lys167 loop region is stable when pS273R binds a ligand and that it likely regulates substrate binding and recognition and precise cleavage of pp220 and pp62 by pS273R. Leucovorin, Carboprost, Protirelin, Flavin Mononucleotide, and lovastatin acid altered the FEL of pS273R, which was consistent with the findings of our MD analysis. The solvent exposure of the pS273R-catalyzed triplet may be impacted by this altered conformational distribution of pS273R, resulting in pS273R inactivation. The pS273R multi-conformation is favored by all five molecules above, and binding to this conformation is more akin to a flexible state of change. After 20 ns of simulation, the average number of H-bonds formed between the pS273R protein and Leucovorin, Carboprost, Protirelin, Flavin Mononucleotide, and Lovastatin Acid supported our hypothesis that Protirelin, Flavin Mononucleotide, and Leucovorin form more durable and continuous H-bonds with pS273R. The polar and non-polar properties of the five compounds above did not significantly differ according to MM/PB(GB)SA, with van der Waals, electrostatic contributions, and non-polar properties favoring the stability of the binding mode and polar properties working against it. These calculations demonstrate the potential of Leucovorin, Carboprost, Protirelin, Flavin Mononucleotide, and Lovastatin Acid to inhibit pS273R activity. In addition, the candidate molecule’s similarity to known pS273R inhibitors also seems to hint at its biological activity. Molecular similarity comparison based on Tanimoto coefficient is widely used in the field of drug design and screening [37]. Extend-connectivity Fingerprints (ECFP) derived from Morgan algorithm are the standard method for structure-activity relationship studies [38]. Comparing the similarities between candidate molecules and known active drugs will help us understand the structure-activity relationship of drugs and explore novel molecular frameworks. 

To date, dozens of potential candidate inhibitors of the new coronavirus have been screened using computer-aided drug design techniques (CADD) [39]. Study of animal viral diseases, such as the highly pathogenic PRRSV (Porcine Reproductive and Respiratory Syndrome) and ASFV [40], using CADD methods is also important. Unlike earlier studies, this study used multi-conformation virtual screening and a method that combined multiple scoring functions to make predictions. In this way, the hit rate of virtual screening can be increased, particularly for target proteins with flexible conformations.

Overall, this study validated the potential of Leucovorin, Carboprost, Protirelin, Flavin Mononucleotide, and Lovastatin to inhibit pS273R activity. The findings support the further evaluation of these drugs as candidates for anti-ASFV treatments.

## 4. Materials and Methods

### 4.1. Sampling and Clustering of Protein Targets Conformations

The Crystal structure of the ASVF pS273R was retrieved from the RSCB database (https://www.rcsb.org, accessed on 20 October 2022). The GROMACS 2022.3 suite was used on a Linux-based workstation [41]. The Amber14sb force field was used to describe the full range of interatomic interactions throughout the simulation [42]. The water model TIP3P was used to simulate the solemnization of the system [43], and sodium and chloride ions were added to simulate the physiological concentration of 0.15 M NaCl. The steepest descent and conjugate gradient methods were used for energy minimization, each system was then balanced with 300 ps NVT (number of isoparticles N, volume V, temperature T) and 300 ps NPT (number of isoparticles N, P, T). The system underwent a long-range electrostatic interaction using the particle-mesh-Ewald (PME) method [44], the linear constraint solver (LINCS) algorithm was used to constrain the bond length of heavy atoms [45]. Short-range interactions were calculated using the Verlet truncation scheme, the cutoff for short-range van der Waals interactions and electrostatic interactions was 14 Å. The system obtained from the above steps was subjected to two steps of energy minimization, the steepest descent method (maximum number of steps 50,000, maximum force < 5.0 Kj/mol), and the conjugate gradient method (maximum number of steps 50,000, maximum force < 2.0 Kj/mol). After completion of minimization, the system was simulated for 300 ps NVT (number of isoparticles N, V, and T) using the Parrinello Danadio Bussi temperature-coupling algorithm. After completion of the NVT [46], a 300 ps NPT balance (number of isoparticles N, P, T) was applied using the Parrinello-Rahman pressure coupling algorithm. After reaching the necessary equilibrium [47], simulations were performed at a constant temperature (300 K) and pressure (1 atm) for 200 ns with a simulation step size of 2 fs.

After the simulation, the RMSD, RMSF, (SPM) of the system were analyzed [48], extraction of conformational distribution during the 200 ns simulation was achieved using TTClust and visualized using PyMol (https://pymol.org/2/, accessed on 30 October 2022) and OriginPro2020 (https://www.originlab.com, accessed on 30 October 2022) [49]. The main conformation of pS273R will be used in the subsequent virtual screening.

### 4.2. Virtual Screening 

The FDA drug structure was downloaded from the e-Drug3D database and saved in the structure data file (SDF) format. The e-Drug3D database contains 2056 structures of FDA-approved compounds, all structurally optimized, and sdf_split.py in Python 3.7 was used to split the compound ensemble and convert it into a separate PDB file. Finally, the compound ligand PDBQT (protein data bank (PDB), partial charge (Q), and atom type (T)) file containing the Gasteiger charges and rotatable bond numbers was created using the prepare_ligand.py script included in MGLTools-1.5.7 [50], in which the -U lps command was used to ensure that the nonpolar hydrogen information was fully recorded in the PDBQT file. Virtual screening of the FDA compound library and pS273R multiple conformations was performed using Watvina. The getbox plug-in in PyMol was used to obtain the coordinates of the recipient docking box with the following parameters: population = 5, ga _ search = 5. The implicit sol command was used to add an implicit water model to approximate solvent action. Three conformational Watvina scores of pS273R were selected, all < −6 kcal/mol. Artificial intelligence-based scoring functions can improve the performance of virtual screening. It was proposed a linear combination of scoring functions called MDock scores that combines Watvina (https:// github.com/biochemistry/watvina, accessed on 25 October 2022), Sfcnn, delta_LinF9_XGB, and RF-Score-VS ligand-receptor docking scoring methods to calculate the score for each ligand [27,51,52]. The calculation method is as follows: P_conf_ in the following formula represents the weight of the conformation, and Ls scores each of the scoring functions for normalized linear docking.

$$\mathrm{MDock}\;\mathrm{Score}=P_{\mathrm{conf}}\sum_{\mathrm{conf}}^{\max\left(\mathrm{conf}\right)}{\left(Ls_{\left|wat\right|}+Ls_{Sfcnn}+Ls_{delta\_LinF9\_XGB\;}+Ls_{\;RF\_Score\_VS}\;\right)}_{\;}$$

For further analysis, we chose the top five molecules based on MDock scoring and used PLIP to examine how the putative molecules interacted with pS273R [53].

### 4.3. Molecular Dynamics Simulations

The top five numerators in the MDock score ranking and molecular dynamics (MD) simulations were performed on the complexes of pS273R protein and its corresponding putative molecules using GROMACS 2022.3. The general amber force field (GAFF) and AM1-BCC charge were used for the treatment of ligand molecules (hypothetical inhibitors) [54]. Parameters and topological files of ligands under GROMACS were generated using Antechamber (AMBER) and Acpype tools. Other than the GAFF of the ligands, the simulated parameters were consistent with 4.1. The RMSD, RMSF, SASA, and number of H-bonds were then calculated, and FEL maps were projected onto the main components PC1 and PC2. Data were visualized using PyMOL, Chimera X, and origin2022.

### 4.4. Binding-Free Energy of the Protein–Ligand Complexes

The MM/PB(GB)SA method is commonly describes the binding stability of protein receptors to small molecule ligands [55], and has been widely used to calculate the binding free energy between proteins and inhibitors. The MM/PB(GB)SA binding free energy was defined as follows:

ΔGbind = Gcomplex − (Gfree-protein + Gfree-ligand)

The gmx_MMBPSA.py was used to calculate the binding free energy of the pS273R protein to its putative inhibitor [56,57], thereby improving the accuracy of the virtual screening results. Considering the convergence of the analog system, we chose the simulation trajectory after 80 ns for each complex that contained a hypothetical inhibitor.

### 4.5. Fingerprint Calculation and Similarity Comparison of Candidate Molecules

The known pS273R inhibitors and drug candidates in Rdkit 2022.9.3 were calculated with ECFP4 fingerprints. Molecular similarity was compared by the Tanimoto distance of the fingerprint. The known pS273R inhibitors included a set of substrate analogues (ZW22, ZW30, and ZW60) and E64. OPTX-1 is not included here because of its larger molecular mass.

## 5. Conclusions

Together, the MD simulations and conformational clustering revealed conformational changes in the pS273R active site, tentatively identifying Thr159-Lys167 as a “gatekeeper” in the catalytic activity of pS273R in the loop region. The function of the ARM region was explained from the perspective of conformational dynamics, which further revealed the mechanism of the conformational motion of pS273R and suggested the possibility of allosteric inhibitors of pS273R. The potential of the candidate drugs for controlling ASFV infection was analyzed using a virtual screening method which contained multi-conformation docking and combinatorial scoring functions. The five selected FDA drugs have good binding affinity for multiple conformations of pS273R and are potential inhibitors of pS273R proteases. Further analysis showed that Leucovorin, Flavin Mononucleotide, and pS273R had good Gibbs binding free energies. Therefore, Leucovorin and Flavin Mononucleotides are perhaps effective anti-ASFV candidates. Further biological evaluations are required to validate the activity of these drugs. This study provides a theoretical basis for screening anti-ASFV therapeutics and repurposing FDA-approved drugs. 

## Figures and Tables

**Table 1:** Watvina docking scores of FDA-approved drugs with Crystal (6LJ9A), Cluster 1, and Cluster 2.

ACS	Watvina Docking Score (Kcal/mol)		
	Crystal (6LJ9A)	Cluster 1	Cluster 2
78110-38-0	−8.3	−6.6	−6.6
58-05-9	−8.8	−7.2	−6.6
32986-56-4	−7	−6.6	−7.5
59-01-8	−7.3	−6.6	−7.9
146-17-8	−9	−7.3	−7
24305-27-9	−7.8	−6.9	−6.5
27025-41-8	−8.4	−6.7	−8.4
83602-05-5	−7.5	−6.6	−7.1
75225-51-3	−7.7	−7.3	−7.1
259218-79-6	−8.1	−6.8	−7
83-88-5	−8.2	−6.7	−7.2
2921-57-5	−8	−6.6	−7.7
551-11-1	−8	−6.7	−6.9
35700-23-3	−7.2	−7	−6.9
745-65-3	−8	−6.6	−7.2
75847-73-3	−7.7	−7.0	−6.5
86541-75-5	−7.5	−7.2	−6.6
139110-80-8	−7.1	−6.6	−7.3
4697-36-3	−7.8	−6.5	−6.6

Note: Only molecules with docking scores < −6.5 Kcal/mol for pS273R were selected.

**Table 2:** ACS numbers, names, and MDock scores of candidate compounds and their interacting residues with Crystal (6LJ9A), Cluster 1, and Cluster 2.

ACS	Compound	MDock Score	Residue in Contact		
			Crystal (6LJ9A)	Cluster 1	Cluster 2
58-05-9	Leucovorin	2.911	THR159, SER162 GLY166, HIS168 ASN187, ARG224 GLN226	THR159, ASN191 GLN226, HIS168 ARG224	THR159, ASN187 ASN191, ARG224
35700-23-3	Carboprost	2.317	THR159, HIS168 ASN187, ASN191 ARG224, GLN226 GLN229	HIS168, THR159 ASN187, ASP160 THR189	HIS168, ASN187 ASN191, ARG224
24305-27-9	Protirelin	2.243	THR159, GLY166 LYS167, ARG224 GLN226, GLN 229	ASN187, THR189 ASN191, SER192 ARG224, GLN229	LYS167, ASN187 ARG224, GLN229
146-17-8	Flavin Mononucleotide	2.174	THR159, HIS168 ASN187, ARG224 GLN226, SER228	HIS168, ASN187 THR189, ASN191 GLN229	ASN187, THR189 ARG224, SER228
75225-51-3	Lovastatin Acid	2.154	ASN191, ARG224 HIS168	THR159, ASP160 ASN191	LYS167, GLN226 SER228, HIS168

**Table 3:** Gibbs binding free energy (Kcal/mol) of the five hypothetical inhibitors with pS273R calculated using MM/GBSA.

	ΔE vdW	ΔE elec	ΔG pol	ΔG nonpol	ΔG Binding
Leucovorin	−29.41 ± 4.05	−154.07 ± 8.72	154.51 ± 7.08	−4.47 ± 0.32	−33.44 ± 1.03
Carboprost	−21.77 ± 4.33	−102.76 ± 7.41	96.99 ± 5.78	−4.17 ± 0.52	−31.71 ± 4.58
Protirelin	−26.61 ± 4.55	−13.70 ± 3.86	18.29 ± 3.47	−3.53 ± 0.52	−25.55 ± 4.34
Flavin Mononucleotide	−33.77 ± 2.91	−140.93 ± 8.05	145.14 ± 8.17	−3.53 ± 0.52	−34.14 ± 2.87
Lovastatin Acid	−22.66 ± 3.01	−95.82 ± 15.36	95.51 ± 12.96	−3.61 ± 0.37	−26.58 ± 7.62

**Table 4:** Gibbs binding free energy (Kcal/mol) of the five hypothetical inhibitors with pS273R calculated using MM/PBSA.

	ΔE vdW	ΔE elec	ΔG pol	ΔG nonpol	ΔG Binding
Leucovorin	−29.41 ± 4.05	−154.07 ± 8.72	152.54 ± 7.16	−3.70 ± 0.16	−34.63 ± 3.36
Carboprost	−21.77 ± 4.33	−102.76 ± 7.41	99.09 ± 6.23	−3.14 ± 0.28	−28.58 ± 3.73
Protirelin	−26.61 ± 4.55	−13.70 ± 3.86	19.14 ± 4.08	−2.67 ± 0.33	−23.84 ± 3.43
Flavin Mononucleotide	−33.77 ± 2.91	−140.93 ± 8.05	145.91 ± 6.48	−3.41 ± 0.12	−32.20 ± 2.88
Lovastatin Acid	−46.4 ± 3.4	−44.2 ± 6.0	54.4 ± 4.5	−4.3 ± 0.3	−27.01 ± 2.9

## Data Availability

The data that support the findings of this study are available from the corresponding author upon reasonable request.

## Supplementary Materials

The following supporting information can be downloaded at: https://www.mdpi.com/article/10.3390/molecules28020570/s1, Figure S1: RMSF plots of pS273R complexes with candidate molecule and free state pS273R; Figure S2: Average structure of pS273R complexes with candidate molecules; Table S1: MDockScore calculation for candidate molecules; Table S2: Complex simulations of the average structure extracted with the RMSD of the initial structure of pS273R(Crystal (6LJ9A),Cluster1 and Cluster2).
