# Supplementary material for: Structural Analysis, Multi-Conformation Virtual Screening and Molecular Simulation to Identify Potential Inhibitors Targeting pS273R Proteases of African Swine Fever Virus
Source: Molecules. 2023 Jan 6;28(2):570. doi: 10.3390/molecules28020570 (PMC9866604; doi:10.3390/molecules28020570)
Supplement: Supplementary file 1 [file molecules-28-00570-s001.zip › Supplementary Figures S1, S2 and Table S2.pdf]

Supplementary Figures S1, S2 and Table S2

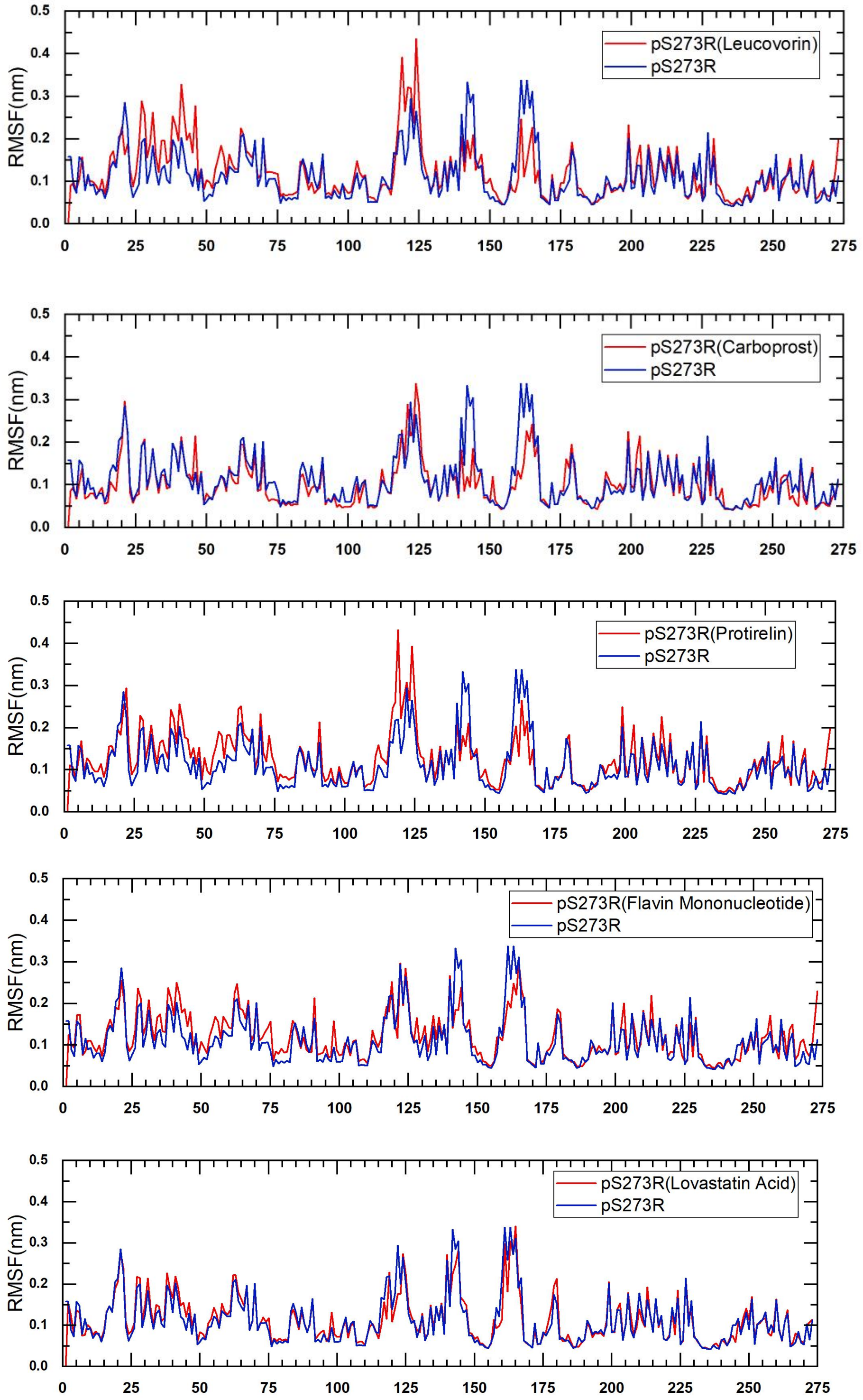

Figure S1 RMSF plots of pS273R complexes with candidate molecule and free state pS273R. RMSF plots of pS273R−Leucovorin, pS273R−Carboprost, pS 273R−Protirelin, pS273R−Flavin Mononucleotide, pS273R−Lovastatin Acid with free state pS273R. The vertical coordinate is the residue number.

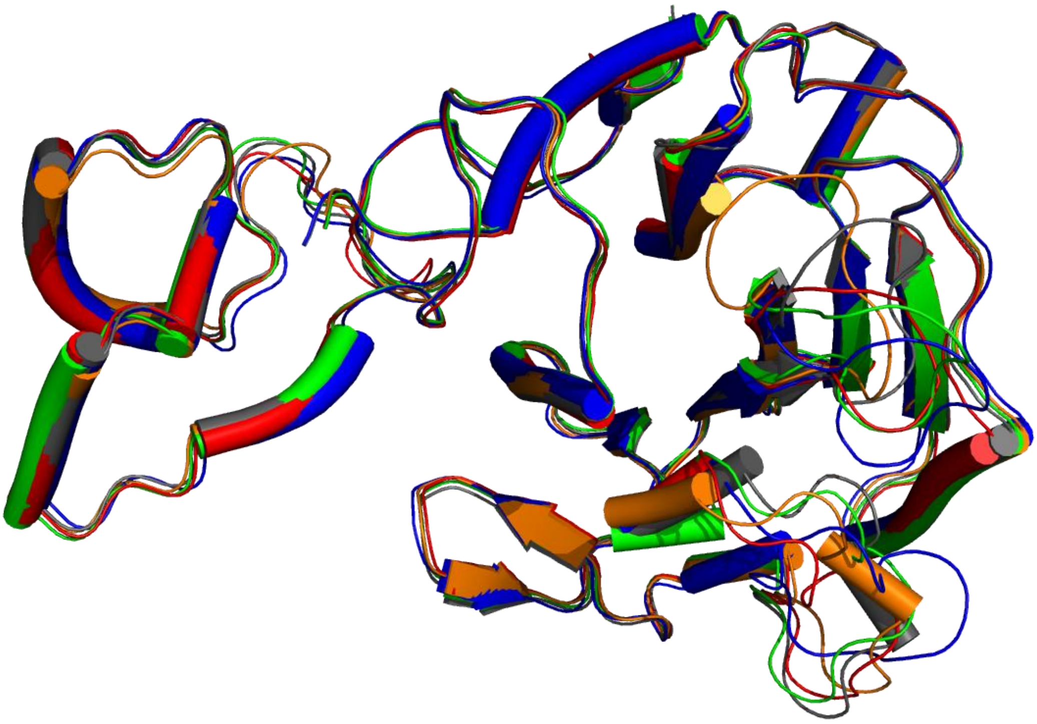

FigureS2 Average structure of pS273R complexes with candidate molecules (Leucovorin, Carboprost, Protirelin, Flavin Mononucleotide and Lovastatin Acid) extracted from molecular simulation trajectories of 20-100 ns (structure containing only pS273R). Red: pS273R−Leucovorin, blue:pS273R−Carboprost, yellow:pS273R−Protirelin, green:pS273R−FlavinMononucleotide. grey: pS273R−Lovastatin Acid

Table S2: Complex simulations of the average structure extracted with the RMSD of the initial structure of pS273R(Crystal (6LJ9A),Cluster1 and Cluster2).

**$pS273R_{lig}Avg$**	**$pS273R_{lig}Avg$ RMSD αC（nm）**		
	**Crystal（6LJ9A）**	**Cluster1**	**Cluster2**
pS273R−Leucovorin	0.772	0.901	1.184
pS273R−Carboprost	1.171	0.962	0.896
pS273R−Protirelin	0.715	0.812	0.950
pS273R−Flavin Mononucleotide	0.833	0.782	0.927
pS273R−Lovastatin Acid	0.693	0.810	1.150
